# Unified Theory of Ultimate Hydrocarbon Recovery for Primary and Cyclic Injection Processes in Ultratight Reservoirs

**DOI:** 10.1038/s41598-019-47099-3

**Published:** 2019-07-24

**Authors:** Michael Cronin, Hamid Emami-Meybodi, Russell T. Johns

**Affiliations:** 1 Department of Energy and Mineral Engineering, The Pennsylvania State University, University Park, PA 16802 USA; 2 EMS Energy Institute, The Pennsylvania State University, University Park, PA 16802 USA

**Keywords:** Natural gas, Crude oil

## Abstract

This paper presents a simple method to estimate ultimate recovery factors (URF) of ultratight reservoirs based on equilibrium by diffusion in which URF is only a function of changes in hydrocarbon density between initial and final states. URF is defined at infinite time and therefore does not depend on the transient behavior. Although URF may not be achievable during the life-cycle of the field development and production, it provides valuable insights on the role of phase behavior. We show that equilibrium phase behavior defines the absolute upper-bound for URF during primary production and explains the poor recovery from shale oil reservoirs compared to the high recovery factor in shale gas reservoirs in a unifying way. Further, we quantify how injected solvent compositions (CH_4_, CO_2_, N_2_, and C_2_H_6_) during huff’n’puff enhanced oil recovery (EOR) improve recovery based on density reduction and compositional dilution, and show that the largest percentage increase in recovery occurs for heavier oils. Our calculations provide a practical means to define the URF from primary production as a function of reservoir fluid composition, temperature, and pressure drawdown. In addition, our calculations articulate incremental URF (IURF) of solvent huff‘n’puff based on net solvent transfer into ultratight rock, which is a key design consideration. The results illustrate that solvent transfer dilutes the hydrocarbons in place, thus maximizing long-term hydrocarbon recovery. Net mass transfer can be improved by enhancing the diffusion of solvent into the matrix based on the huff‘n’puff design parameters including solvent composition, drawdown pressure, and the net amount of solvent injected based on optimal frequency and cycle duration.

## Introduction

Hydrocarbon recovery factors (fraction of original oil in place, OOIP, or original gas in place, OGIP) in unconventional reservoirs vary as a function of fluid type; typically less than 10% for oils^1,2^, 30–70% for gases^3^, and somewhere in between for gas condensates. The enormous resource potential in such reservoirs and their relative ubiquity around the world^4^ motivate two desires; first, to understand why primary recovery changes significantly between fluid compositions and reservoirs, and second, to understand how much recovery can be improved based on adjusting drawdown during primary production or using a solvent-based (CH_4_, CO_2_, N_2_, C_2_H_6_, etc.) process.

In primary production of conventional reservoirs, hydrocarbon is often recovered based on fluid transport caused by pressure drawdown at the well. The unconventional reservoir literature has emphasized the importance of the stimulated fracture network (sometimes referred to as stimulated rock volume, SRV^5^), well spacing, and “sweet spots” to improve recovery. These correlations can provide insight into drilling locations and expected recoveries, but they are not fundamentally based on the underlying physics and require significant historical data. Instead, maximum recovery (at infinite time) can be easily quantified by the density reduction between initial and producing conditions, which is independent of fracture-matrix contact area and transport rates^6^.

Solvent-based methods have been proposed in unconventional reservoirs for both enhanced oil recovery (EOR)^7–12^ and enhanced gas recovery (EGR) applications for condensates^13–16^ based on cyclic solvent injection, i.e., “huff‘n’puff”. However, many of the published studies (experimental or numerical) approach the problem from a time-dependent perspective instead of providing clear insight into the theoretical upper-bound recovery values. Theoretical upper-bound values are important because they could quickly allow designers to screen huff‘n’puff (HnP) design parameters (injection pressure, solvent composition, solvent amount injected, cycle number and duration) for a given reservoir composition. Furthermore, knowing how to increase the ultimate recovery factors could yield economic increases in the transient recovery. There needs to be a clear framework to approach reservoir development as a unified process spanning primary production and solvent-based EOR/EGR for different fluid compositions.

Miscible gas injection based on the multi-contact-miscibility (MCM) process requires knowledge of which tie line controls miscibility (that is, when a tie line in the two-phase region obtains zero length first). Miscibility attained in this manner results from interaction of advective transport (flow governed by relative permeability) and phase behavior such that the forward contact (oil tie line), backward contact (gas tie line), or a cross-over tie line controls miscibility^17,18^. In an ultratight reservoir matrix, however, miscibility becomes a function of solubility because advection-dominated flow conditions required for the MCM process are no longer relevant^6^. Instead, transport within a tight matrix is anticipated to be a diffusion-dominated process^6,19^. Concentration gradients within the matrix near the matrix-fracture interface control mass transport in these reservoirs for the primary production and HnP process due to the significant contact area created during hydraulic fracturing between the matrix and fracture network.

A unified theory applicable to primary recovery for any fluid type and for any cyclic injection sequence is needed to quantify practical expectations for the maximum ultimate recovery factor achievable. In this paper, we first develop such a unified theory based on diffusion principles, where equilibrium is ultimately achieved between an initial and final state. We evaluate ultimate mass recovery factors for the *n*-alkane series as a compositional proxy for a range in unconventional reservoir compositions. Next, we compare the potential use of four different solvents (CH_4_, CO_2_, C_2_H_6_, and N_2_) to enhance ultimate recovery factor for various reservoir compositions, reservoir conditions, and design parameters (amount of solvent injected and producing pressure). We then evaluate recovery (primary and HnP) using several Eagle Ford compositions. Finally, we discuss the theoretical and practical implications of these results for a multiple-cycle HnP process.

## Theory and Methods

In this section, we first develop the underlying theory of ultimate recovery for the primary and HnP processes. We then present a sensitivity analysis using a compositional proxy that captures a range of reservoir fluid types from liquid-like to vapor-like.

### Theory

Diffusion should equilibrate overall compositions if given sufficient time. Thus, ultimate hydrocarbon mass recovery is strictly a function of the equilibrium hydrocarbon pressure-volume-temperature (PVT) behavior (phase densities, compositions, and saturation) observed at initial (*t* = 0) and final (*t* → ∞) reservoir conditions. In a reservoir system with *n*_*c*_ components and *n*_*p*_ fluid phases, the ultimate mass recovery factor for each component, *URF*_*i*_ [fraction of original component *i* mass in place], is,

1 $$UR{F}_{i}=1-{C}_{i,F}/{C}_{i,I}=1-\sum _{j=1}^{{n}_{p}}{({S}_{j}{\rho }_{j}{\omega }_{ij})}_{F}/\sum _{j=1}^{{n}_{p}}{({S}_{j}{\rho }_{j}{\omega }_{ij})}_{I},$$

where *S*_*j*_ [−] is phase saturation, *ρ*_*j*_ [lbm/ft^3^] is phase density, *ω*_*ij*_ [−] is the mass fraction for component *i* in phase *j*, *C*_*i*_ [lbm/ft^3^] is the total mass concentration for component *i* in the pore space.

Subscripts *I* and *F* refer to initial (*t* = 0) and final (*t* → ∞) conditions in the reservoir for pressure *p* [psia], temperature *T*_*res*_ [°F], and overall mole fraction vector $\overrightarrow{z}=[{z}_{1},{z}_{2},\,\ldots ,\,{z}_{{n}_{c}}]$ subject to $\sum _{i}^{{n}_{c}}{z}_{i}=1.0$. Equation (1) allows for any number of phases to be present in the reservoir at initial or final conditions, but assumes no-flux outer boundaries, constant pore volume, spatially uniform initial pressure and temperature distributions, and no gravity effects.

### Recovery processes

Equation (1) is a general equation for URF that applies to primary and/or HnP processes. Equation (1) shows that ultimate recovery for hydrocarbon components will be improved by any process capable of reducing *C*_*i*,*F*_ below the initial reservoir concentration^19^.

#### Primary production

Primary recovery results mainly from the density reduction (fluid expansion) that occurs in the reservoir as pressure declines. Thus, increasing the amount of pressure drawdown (*p*_*I*_ − *p*_*wf*_) during primary production will increase the ultimate recovery factor (attained after an infinite period of production). Equation (1) is used to estimate the ultimate mass recovery [fraction of original component *i* mass] following primary production, $UR{F}_{i,P}=1-{C}_{i,P}/{C}_{i,I}$, where subscript *P* replaces *F* to denote “primary”. *C*_*i*,*P*_ [lbm/ft^3^] is the mass concentration of component *i* at equilibrium conditions following an infinitely-long period of primary production (${T}_{res},\,{p}_{wf},\,{\overrightarrow{z}}_{P},\,t\to \infty$). The composition ${\overrightarrow{z}}_{P}={[{z}_{1},{z}_{2},\ldots ,{z}_{n}]}_{P}$ is the overall equilibrium composition in the reservoir following primary production. ${\overrightarrow{z}}_{P}$ will be constant through the primary recovery process as long as all components can move coherently through the nm-sized pores. However, filtration^20^, size exclusion^21^, and adsorption^21^ effects may selectively hinder component transport within small pores or those lined with organic matter. Chromatographic separation^22^ could also be important in tight/shale gas reservoirs.

Equation (1) can be simplified when the overall composition of the fluid is constant during the primary recovery process and the pressure does not go below the bubble point. Confinement tends to suppress bubble point pressure within nanopores^23,24^. In this case, the primary ultimate recovery factor, *URF*_*P*_, is controlled by the initial (*ρ*_*I*_) and final (*ρ*_*F*_) fluid densities within the reservoir, where *URF*_*P*_ = 1 − *ρ*_*I*_/*ρ*_*F*_.

#### Huff‘n’puff (cyclic solvent) process

**Figure 1:**
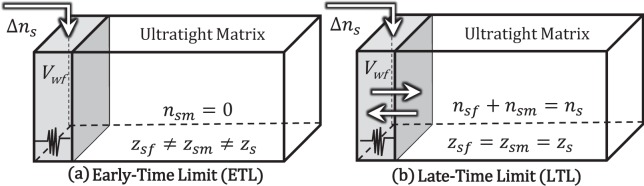
Conceptual tank models for the (**a**) early-time limit (ETL) and (**b**) late-time limit (LTL). In the ETL, solvent (Δ*n*_*s*_, [lbmol]) is injected into *V*_*wf*_ and subsequently produced back in the huff with no mass-transfer between *V*_*wf*_ and the ultratight matrix. In the LTL, perfect mixing is achieved (*z*_*sf*_ = *z*_*sm*_ = *z*_*s*_) by diffusion during the soaking period (as *t* → ∞) before starting the huff. *n*_*sf*_, *n*_*sm*_, and *n*_*s*_ are the amounts of solvent [lbmol] present in the *V*_*wf*_, ultratight matrix, and the entire reservoir, respectively, and *z*_*sf*_, *z*_*sm*_, and *z*_*s*_ are the corresponding solvent mole fractions.

A second way to increase URF is the use of a Huff‘n’Puff (HnP) process to reduce *C*_*i*_ by decreasing *ρ*_*j*_ terms (fluid expansion) and decreasing *ω*_*ij*_ (selective dilution of hydrocarbon components) in the reservoir. Solvent-hydrocarbon mixing during the huff and soak causes a local change in fluid composition in the fracture network and adjacent matrix blocks contacting the fracture. We consider two conceptual limits for solvent-hydrocarbon mixing. The lower limit, which we refer to as the early-time limit (ETL), considers perfect solvent mixing throughout the wellbore and fractures by a combination of advection (Darcy’s law) and diffusion. Mixing in the early-time volume is not instantaneous, but the ETL assumes no mass-transfer between ultratight matrix and the early-time volume owing to the large conductivity contrast. The upper limit, which we refer to as the late-time limit (LTL), considers perfect solvent mixing throughout the entire reservoir. After a long period of time (*t* → ∞), diffusion will bring the ultratight matrix into equilibrium with the early-time volume. Figure 1 schematically illustrates the ETL and LTL tank models.

Equation (1) is used to determine the URF’s for the HnP process. The fluid composition used in Eq. (1) is assumed to be the equilibrium composition obtained from material balance at the end of the huff and soak period, which is determined by the mass of solvent injected during the huff and the total mass of hydrocarbon remaining in the reservoir after primary. The final densities and compositions are then used in Eq. (1) to determine the ultimate mass recovery [fraction of original component *i* mass] following HnP, $UR{F}_{i,H}=1-{C}_{i,H}/{C}_{i,I}$, where subscript *H* replaces *F* to denote “huff”.

In the case of multiple HnP cycles, one must account for the mass of fluid and components (a mixture of hydrocarbon and solvent) produced back during each puff cycle to determine the net amount of solvent (*n*_*s*_ [lbmol]) and remaining hydrocarbon (*n*_*hc*_ [lbmol]) in the reservoir,

2a $${n}_{hc}=\sum _{i=1}^{{n}_{c}}{n}_{i,I}-\sum _{i=1}^{{n}_{c}}{n}_{i}^{prod},$$

2b $${n}_{s}={n}_{s}^{inj}-{n}_{s}^{prod},$$

where *n*_*i*,*I*_ [lbmol] and ${n}_{i}^{prod}$ [lbmol] are the initial and produced amounts of each component, and ${n}_{s}^{inj}$ [lbmol] and ${n}_{s}^{prod}$ [lbmol] are the injected and produced amounts of solvent.

We assume equilibrium conditions and compute recovery using compositions from a material balance as a function of the net mole fraction of solvent in the reservoir, *z*_*s*_, given as *z*_*s*_ = *n*_*s*_/(*n*_*s*_ + *n*_*hc*_). Values for *z*_*s*_ are physically restricted between 0 and 1 [mole fraction]. These limiting values span the continuum between zero solvent injection (*z*_*s*_ = 0, primary only) and perfect hydrocarbon dilution/replacement with solvent (*z*_*s*_ = 1) in the reservoir. The practical upper-bound for *z*_*s*_ from HnP is much less than unity because operating constraints restrict how much solvent can be injected during a given huff cycle. Thus, multiple cycles are typically needed to increase *z*_*s*_, although incremental increases in *z*_*s*_ from each additional cycle likely become less. The importance of *z*_*s*_ to recovery argues also to increase the injection pressure to its maximum field value.

The values used in Eq. (2) come from either production data or analytical/numerical simulation. Therefore, the actual masses of components produced and injected during each HnP cycle at a well(s) could also be used to directly calculate the expected URF after all HnP cycles are complete. In this paper, we first consider only one HnP cycle to illustrate the fundamentals underlying the recovery process. In the discussion, we extend observations and findings to multiple cycle HnP.

By design, solvent IURF is maximized in the LTL and minimized in the ETL. The performance of a real HnP cycle is expected to fall in between these two limits. We quantify HnP performance based on incremental ultimate recovery factor IURF_i_, [fraction of original component *i* mass] because EOR and/or EGR operations are judged according to the economic value of incremental hydrocarbon production compared with the reference case (recovery without EOR/EGR). Accordingly, the solvent IURF corresponding to the ETL (*IURF*_*i*,*ETL*_) is calculated by,

3 $$IUR{F}_{i,ETL}=\frac{{V}_{wf}}{P{V}_{Total}}\frac{{C}_{i,P}-{C}_{i,H}^{ETL}}{{C}_{i,I}},$$

where ${C}_{i,H}^{ETL}$ is the total mass concentration for component *i* based on the ETL composition at puff conditions and *PV*_*Total*_ [ft^3^] is the pore volume of the entire reservoir.

Lumping the wellbore and fractures together into a cup-mixed tank with pore volume *V*_*wf*_ [ft^3^] is reasonable for ultratight reservoirs that act on completely different time scales. Highly permeable layers (e.g. carrier beds) connected to the fractures could, perhaps, also be lumped into *V*_*wf*_. One can then calculate the minimum injected solvent (Δ*n*_*s*_, [lbmol]) required to pressurize *V*_*wf*_ to a target bottom-hole injection pressure (*p*_*H*_ [psia]) using a compositional volume balance method^25,26^,

4 $$VBE={V}_{wf}({p}_{H})-{V}_{fluid}({p}_{H},{T}_{res},{\overrightarrow{z}}_{wf})=0$$

where *VBE* [ft^3^] is the volume balance error (pore volume − fluid volume), ${\overrightarrow{z}}_{wf}=[{z}_{1},{z}_{2},\ldots ,{z}_{n}]$ [mole fraction] is the cup-mixed overall composition in the fractures, *V*_*fluid*_ [ft^3^] is the water, oil, and gas phase volumes at the specified pressure, temperature, and composition.

Counter-diffusion occurs during the huff and soak because solvent diffuses from fractures into matrix while hydrocarbon simultaneously diffuses into the fractures^6,19,26^. However, in this study, we assume a constant pore volume and ignore mass transfer between *V*_*wf*_ and ultratight matrix when using Eq. (4). Furthermore, *VBE* was implemented when calculating the amount of injected solvent required to pressurize the wellbore-fracture volume to a target pore pressure in the absence of diffusive mass transfer between matrix and *V*_*wf*_. The wellbore-fracture pore volume is fixed when pressure is specified. However, the volume of the hydrocarbon-solvent mixture depends on the mass of injected solvent. The amount of injected solvent is iteratively improved via Newton-Raphson with respect to *VBE* until volume balance is achieved, i.e., $|VBE/{V}_{wf}| < {10}^{-6}$. In other words, the amount injected was iteratively improved using pore volume minus fluid volume as the objective function to minimize in a Newton-Raphson update based on ${\rm{\Delta }}{n}_{s,new}={\rm{\Delta }}{n}_{s,old}-VBE({\rm{\Delta }}{n}_{s,old})/VBE^{\prime} ({\rm{\Delta }}{n}_{s,old})$, where *VBE*′ is the derivative of the *VBE* with respect to the moles of injected solvent (Δ*n*_*s*_). We conducted a sensitivity study and found that relative error asymptotically stabilizes for any convergence criterion less than 10^−4^. We chose 10^−6^ to be conservative and align with typical reservoir simulation convergence criteria.

The solvent IURF corresponding to the LTL (*IURF*_*i*_) is calculated by,

5 $$IUR{F}_{i}=\frac{{C}_{i,P}-{C}_{i,H}^{LTL}}{{C}_{i,I}},$$

where *C*_*i*,*P*_ and ${C}_{i,H}^{LTL}$ are the only terms that can be modified in field applications because *C*_*i*,*I*_ is fixed. As discussed earlier, *C*_*i*,*P*_ is decreased using pressure-drawdown. ${C}_{i,H}^{LTL}$ is decreased further during HnP by changing the composition of fluids in the reservoir (compositional dilution). Thus, solvent composition, amount of solvent, and pressure drawdown (initial reservoir pressure minus wellbore pressure) are the three adjustable factors explored in this paper. The difference between Eqs (3) and (5) is the pore volume fraction (*V*_*wf*_/*PV*_*Total*_) multiplier and the composition vector used to compute mass concentrations in the huff. We assume *V*_*wf*_/*PV*_*Total*_ = 0.01 [−] based on flowback analysis^27^.

### Compositional proxy system

To demonstrate how the proposed unified theory can explain ultimate hydrocarbon recovery, we use a normal alkane (*n*-alkane) series from C_1_ to *n*-C_25_ as a compositional proxy to continuously represent a range of real reservoir compositions from natural gas (light alkanes) to oil (heavy alkanes). This is because real oils contain a complex mixture of paraffins, naphthenes, and aromatics making it difficult to draw any conclusions without simplifying assumptions. We chose *n*-C_25_ as the upper end of the series because production becomes minimal for long hydrocarbons through the small pore-throats in shale reservoirs. This conclusion is supported by comparing the composition of wellstream samples versus those obtained via solvent extraction of crushed core materials^28^.

**Figure 2:**
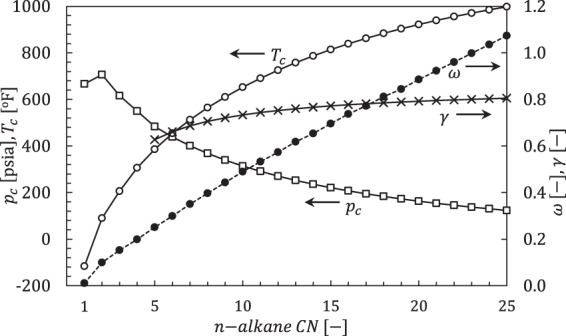
Critical properties and specific gravity versus *n*-alkane carbon number (*CN*) based on experimental values^31^ and correlations^31,33,34^.

Experimental property values do not exist for pure *n*-paraffins heavier than approximately *n*-C_25_^29^. We supplemented experimental data^30^ using the Magoulas-Tassios^31^ critical property correlations (*T*_*c*_, *p*_*c*_, *ω*), which are suitably accurate^32^ for the *n*-alkanes we investigate. We used Whitson’s best-fit^33^ to Yarborough’s correlation^34^ to obtain standard condition specific gravity (*γ* [−]) values for liquid n-alkanes and compute Péneloux volume shift^35^ parameters for the Peng-Robinson^36^ equation of state (PR EoS). Binary interaction parameters were from Whitson^29^ for CO_2_- and N_2_-hydrocarbon pairs and the Chueh-Prausnitz equation^37^ for C_1_-C_7_+ pairs. All other hydrocarbon-hydrocarbon pairs were zero. Figure 2 shows these input parameters as a function of carbon number (*CN*). This approach to the input parameters gives consistent and continuous values. Experiments^30^ show that critical pressures monotonically decrease for *n*-alkanes as *CN* increases (Fig. 2). Ethane is the exception because a lack of compliance in the single carbon-carbon bond prohibits ethane from packing as efficiently as the heavier alkanes containing multiple carbon-carbon bonds^38^.

## Results

In this section, we evaluate changes in primary ultimate recovery factor (URF_P_) as functions of reservoir composition (*n*-alkane), reservoir pressure, drawdown pressure, and reservoir temperature for the compositional proxy system as well as Eagle Ford fluid. Next, we repeat the analysis for HnP where we include the effects of solvent composition and the amount of injected solvent.

### Compositional proxy system

**Figure 3:**
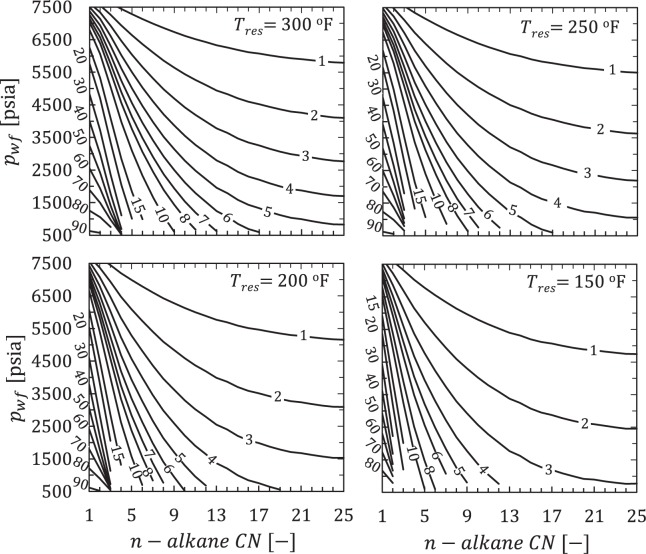
Contour plot of URF_P_ [% of original HC mass] values as a function of primary producing pressure and reservoir composition (pure *n*-alkane) for a fixed initial reservoir pressure of 8000 psia but different reservoir temperatures. Small irregularities in the contours for *CN* > 12 are an artifact of combining multiple independently-derived critical property correlations. Note that only contour values of 1, 2, 3, 4, 5, 6, 7, 8, 10, 15, 20, 30, …, 80, 90 are drawn and labeled as shown.

Figure 3 shows the effect of temperature on primary ultimate recovery factor (URF_P_) versus *n*-alkane CN as *p*_*wf*_ is lowered from *p*_*wf*_ = *p*_*i*_ − *500* = 7500 to *p*_*wf*_ = 500 psia at *T*_*res*_ = 150, 200, 250, and 300°F. Contours were generated by successively solving Eq. (1) at every integer (pure *n*-alkane) *CN* and 5 psi increment for *p*_*wf*_.

Ultimate recovery dramatically improves whenever an initially liquid-like (L) composition becomes more vapor-like (V) or enters the L-V region (vapor pressure curve if pure-component) at final conditions (*p*_*wf*_, *T*_*res*_). Closely-spaced near vertical URF_P_ contours denote the transition between vapor- to liquid-like compositions as *n*-alkane *CN* increases at fixed *p*_*wf*_. The progressive flattening of URF_P_ contours in Fig. 3 confirms the transition from V to L with increasing *CN*. Closely-spaced horizontal URF_P_ contours correspond to crossing a pure-alkane vapor pressure curve at fixed *CN*. Due to higher compressibility in vapor-like phases versus liquid-like phases, the calculated URF_P_ values (which are based on density reduction) are in agreement with our qualitative expectations from compressibility trends.

**Figure 4:**
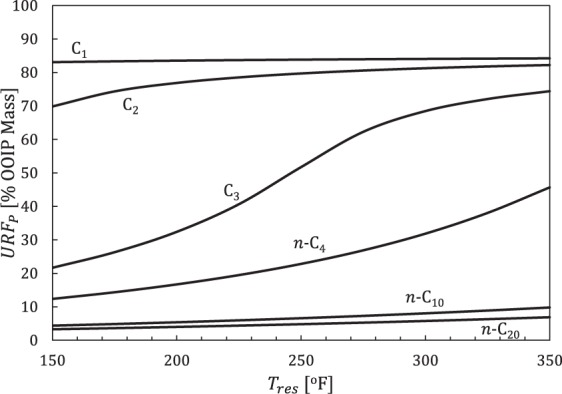
URF_p_ versus temperature for selected *n*-alkanes with *p*_*I*_ = 8000 and *p*_*wf*_ = 1000 psia.

URF_P_ values systematically improve for all *n*-alkanes (C_1_ through n-C_25_) with increasing pressure drawdown (*p*_*I*_ − *p*_*wf*_) and monotonically decrease as *n*-alkanes become heavier (larger *CN*) at any *T*_*res*_ value (Fig. 3) within the pore pressure range of interest. Figure 4 illustrates the variation in URF_P_ with respect to *T*_*res*_ for a few representative compositions (C_1_ for gas, C_2_, C_3_, and *n*-C_4_ for gas-condensate, *n*-C_10_ for volatile oil, and *n*-C_20_ for black oil) at constant *p*_*I*_ = 8000 and *p*_*wf*_ = 1000 psia.

The results in Fig. 4 show that URF_P_ systematically improves with increasing temperature and decreasing *CN* (recall *MW* ≈ 14*CN* + 2). In addition, URF_P_ is more sensitive for C_2_, C_3_, and *n*-C_4_ with respect to temperature than the other compositions, a result shown as well in Fig. 3.

**Figure 5:**
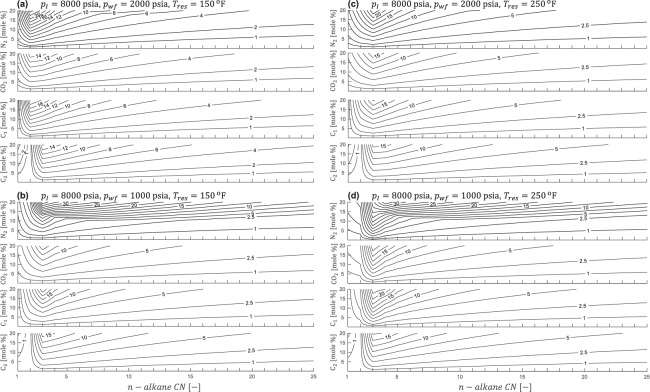
IURF [% OOIP mass] contour plots for solvent HnP. The y-axis is equilibrium net solvent content in the reservoir [*z*_*s*_, mole %] and the x-axis is the reservoir composition proxy (pure *n*-alkane *CN*). IURF = 0 for all solvents when *z*_*s*_ = 0.

Next, we compare the incremental ultimate recovery factor (IURF) using C_1_, C_2_, CO_2_, and N_2_ as the injected solvent at different *T*_*res*_ (150 and 250°F) and *p*_*wf*_ (2000 and 1000 psia) values while keeping the initial reservoir pressure constant at 8000 psia **(**Fig. 5**)**. IURF is calculated using the late-time limit (LTL) for solvent mixing and the assumption that primary and puff periods have the same *p*_*wf*_. The axes correspond to the amount of solvent retained in the reservoir with perfect mixing (*z*_*s*_) versus pure *n*-alkane *CN*. For practical considerations, we restricted *z*_*s*_ to a maximum of 20 mole % since perfect hydrocarbon dilution/replacement (*z*_*s*_ = 100 mole %) will never be reached.

Based on our previous discussion of recovery theory, the solvent IURF analysis in Fig. 5 captures the recovery behavior as the solvent mole fraction approaches its conceptual limits. Solvent IURF is zero for *z*_*s*_ = 0 mole % (primary only) and approaches large finite values as *z*_*s*_ tends to 100 mole %. Solvent IURF must be zero when solvent and *n*-alkane compositions are identical, which is the case for the C_2_ (asymptote of 0.0% for *CN* = 2) and C_1_ (bounding asymptote of 0.0% for *CN* = 1) cases. Closely-spaced horizontal IURF contours in Fig. 5b,d reveal the pronounced vaporization effect of nitrogen solvent on *n*-alkanes that would ordinarily be liquid-like at the given conditions (*p*_*wf*_, *T*_*res*_) but are instead vapor-like (or a two-phase mixture) after the introduction of solvent. Propane (*CN* = 3) broadly experiences the greatest absolute improvement to recovery factor at any given *z*_*s*_ and solvent composition based on finding $\partial /\partial CN[IURF({z}_{s},CN,{p}_{I},{p}_{wf},{T}_{res})]=0$ from graphical analysis (Fig. 5). However, this does not mean that reservoirs with propane-like compositions are “optimal” candidates for solvent HnP.

A practical question is what solvent will be the “best” to inject into a reservoir and how much solvent should be injected. Solvent IURF performance is a function of pressure, temperature, original fluid composition, and the amount and type of solvent injected.

In the context of EOR/EGR, a design is considered “optimal” when it maximizes project economics^17^. On an equal energy content basis, liquid hydrocarbons are more commercially valuable than gaseous hydrocarbons, something which is referred to as the “liquid premium”. Primary recovery factors are very large in the first place for light *n*-alkanes (Figs 3 and 4), which makes HNP less commercially advantageous. In contrast, even a very modest solvent IURF (~1–2% OOIP Mass) would constitute an enormous relative improvement over primary alone (URF_P_ ~3% OOIP Mass) in a heavy oil (Figs 3 and 5). Therefore, the (IURF/URF_P_) ratio may provide a quick screening tool for a more formal economic-based assessment.

**Figure 6:**
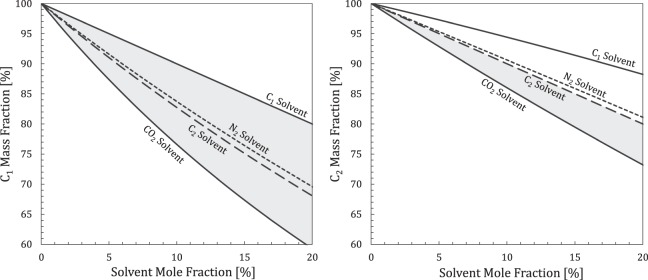
Mass fraction of (**a**) C_1_ and (**b**) C_2_ in a hydrocarbon-solvent mixture versus solvent mole fraction for different solvents, C_1_, N_2_, C_2_, and CO_2_. Compositional dilution (gray-filled region) occurs rapidly for C_1_ with all solvents because the mass fraction of C_1_ changes faster than solvent mole fraction for dilute mixtures. In contrast, rapid compositional dilution only occurs for dilute C_2_-solvent mixtures using CO_2_ due to MW effects.

In a single-phase mixture, the *ρω*_*i*_ product yields component mass concentration. The introduction of solvent initiates two competing processes; compositional dilution (improves solvent IURF by decreasing *ω*_*i*_) and mixing-induced density increases (reduces IURF by increasing *ρ*). The compositional dilution effect occurs whenever the solvent MW is larger than the hydrocarbon MW, which causes *ω*_*hc*_ to decrease faster than *z*_*s*_ increases as solvent is added, which is readily apparent for C_1_ hydrocarbon (Fig. 6a). However, this effect is limited to *CN* < 3 because the MW of *n*-alkanes (14*CN* + 2) quickly exceeds the solvents (Fig. 6b).

If there were no volume change on mixing, compositional dilution would entirely explain differences in IURF among solvents. However, real fluids experience some volume change on mixing due to changes in the density and number of phases. Thus, solvent IURF performance for any hydrocarbon composition is not strictly ordered based on the solvent MW at a given solvent mole fraction and pressure/temperature. Instead, the controlling factor for IURF is the equilibrium phase density (and the number of phases) at the puff conditions. Using C_1_ hydrocarbon as an example, Fig. 6b shows IURF with C_2_ > CO_2_ > N_2_ instead of CO_2_ > C_2_ > N_2_ as predicted by Fig. 6a. C_2_ and CO_2_ solvent behave similarly across the *n*-alkane range. However, N_2_ becomes dominant at lower pressures for heavy *n*-alkanes due to strong vaporizing effects (closely spaced horizontal solvent IURF contours in Fig. 5).

### Eagle Ford case study

We selected the Eagle Ford (South Texas, USA) for two reasons. First, the Eagle Ford spans the entire PVT window from dry-gas, wet-gas, retrograde condensate, volatile oil, to black oil with CH_4_ mole fractions ranging from 72–38%^39^. Second, the Eagle Ford is one of the most prolific/actively developed ultra-tight oil reservoirs in North America with the extensive literature.

In this section, we first analyze primary ultimate recovery factor (URF_P_) values for Eagle Ford compositions ranging from dry gas, lean condensate, rich condensates, to oils reported by Orangi *et al*.^40^. *T*_*res*_ values are [237, 266, 285]°F as oil volatility increases, [295, 318, 310, 303]°F as condensate richness increases, and 352°F for dry gas. Whitson and Sunjerga^39^ reported *T*_*res*_ values between 250 and 330°F for similar Eagle Ford fluids.

**Figure 7:**
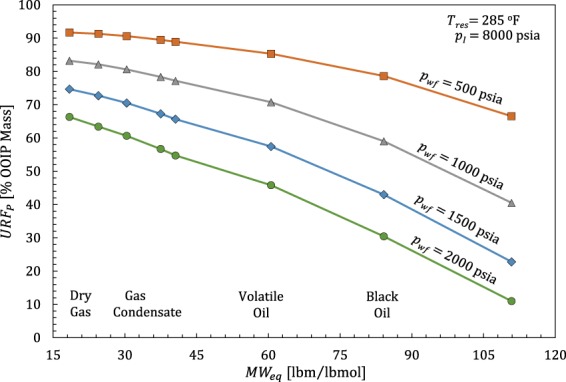
Primary URF [OOIP mass %] versus equivalent hydrocarbon molecular weight for Eagle Ford compositions^40^ with *p*_*wf*_ = 500 psia (squares), 1000 psia (triangles), 1500 psia (diamonds), and 2000 psia (circles). URF_P_ values are calculated assuming volumetric behavior during constant-composition-depletion (CCD) with uniform initial pressure (*p*_*I*_ = 8000 psia) at *T*_*res*_ = 285°F.

Results for pure n-alkanes (Figs 3 and 4) demonstrate that URF_P_ is sensitive to reservoir temperature and initial pressure. Therefore, we standardized reservoir conditions (*p*_*I*_ = 8000 psia, *T*_*res*_ = 285°F) to reflect the oil and condensate averages and eliminate all factors unrelated to fluid composition and producing pressure when comparing URF_P_ values **(**Fig. 7**)**. We use equivalent hydrocarbon molecular weight, $M{W}_{eq}={\sum }^{}{z}_{i}M{W}_{i}/{\sum }^{}{z}_{i}$ [lbm/lbmol], to express fluids as single points in hydrocarbon composition space (the summation excludes non-hydrocarbon components).

Figure 7 shows that URF_P_ values are inversely related to *MW*_*eq*_. Recovery improves for all compositions with increasing drawdown (*p* − *p*_*wf*_), but heavier compositions are more sensitive due to lower fluid compressibilities. Practical (time-dependent) recovery factor (RF) values will be lower due to preferential production of lighter components, limited production timeframes, and un-accessed reservoir volume.

**Figure 8:**
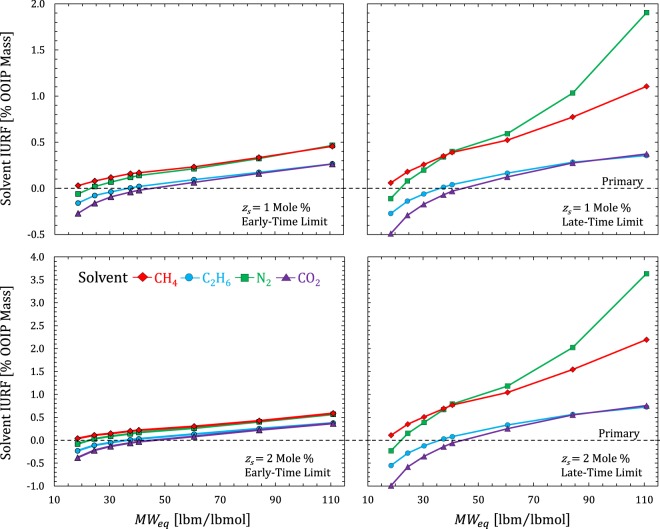
Calculated solvent IURF for Eagle Ford fluid compositions^40^ at two different solvent mole fractions (*z*_*s*_ = 1% and 2%) in the ETL and LTL. RF calculations assume ${V}_{wf}/P{V}_{Total}$ = 0.01 [–], a uniformly depleted reservoir from primary production before injection (*p* = *p*_*wf*_), constant producing pressure (*p*_*wf*_ = 2000 psia) during the puff and constant initial reservoir pressure *p*_*I*_ = 8000 psia at *T*_*res*_ = 285 ^o^F.

Next, we evaluate and compare the HnP performance of four different solvent compositions; CH_4_, C_2_H_6_, CO_2_, and N_2_, to improve the URF beyond primary values. We express improvement based on incremental ultimate recovery factor, IURF [OOIP mass %], summed over all components ($IURF=\sum _{i}^{{n}_{c}}IUR{F}_{i}$). In Fig. 8, we compare primary solvent IURF [OOIP Mass %] for a single cycle of solvent injection in the early-time limit (ETL) defined by Eq. (3) and the late-time limit (LTL) defined by Eq. (5). In practice, the field-scale HnP process is designed based on injecting a fixed volume of solvent or injecting solvent at a fixed maximum bottom hole pressure^26^. The results show that IURF is proportional to the total amount of solvent injected. Positive IURF values indicate an overall reduction in density (successful compositional dilution) while negative IURF values indicate that density is higher in the puff than it was during primary. Incremental recovery may be positive for individual components (*IURF*_*i*_ > 0) even if the overall IURF is negative. C_1_ was beneficial to recovery for all Eagle Ford compositions and the heavier MW solvents (CO_2_ and C_2_) were generally detrimental for the condensate compositions. N_2_ benefited almost all compositions. However, N_2_ was slightly inferior to C_1_ for all compositions except the lowest volatility (gas oil ratio (GOR) = 500 SCF/STB). In general, the oil compositions benefited from using any solvent. Larger slugs are preferred whenever additional solvent tends to improve recovery.

**Figure 9:**
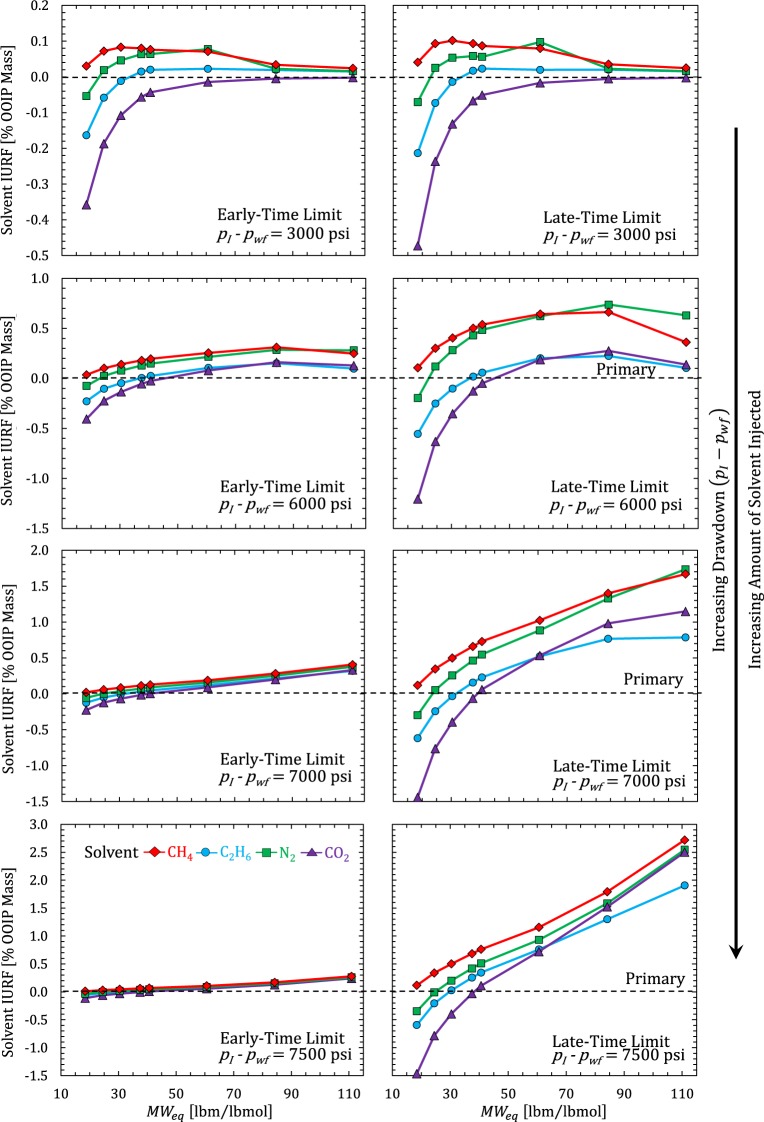
Solvent IURF [% OOIP Mass] versus MW_eq_ [lbm/lbmol] for Eagle Ford fluids based on equal pressurization of *V*_*wf*_ in the huff (*p*_*H*_ = 8000 psia) at two mixing limits (ETL and LTL) and several drawdown values (*p*_*I*_ − *p*_*wf*_). Calculations again assume ${V}_{wf}/P{V}_{Total}$ = 0.01 [–], a uniformly depleted reservoir from primary production before injection (*p* = *p*_*wf*_), *p*_*I*_ = 8000 psia at *T*_*res*_ = 285 ^o^F, and a constant producing pressure during the puff.

In Fig. 9, we compare solvent IURF performance in the early-time and late-time limit based on the same pressure reached at the end of the huff, but assuming different levels of primary reservoir pressure depletion before starting injection (*p*_*I*_ = 8000 psia, *p*_*I*_ − *p*_*wf*_ = 3000, 6000, 7000, and 7500 psi). The total amount of solvent injected progressively increases with drawdown because there is more compressive storage within the early-time volume. In addition, the introduction of solvent tends to increase the fluid compressibility, which allows further amounts of solvent to be injected. Total injected solvent is highest for the gas-like compositions and lowest for the heavy oil compositions.

**Figure 10:**
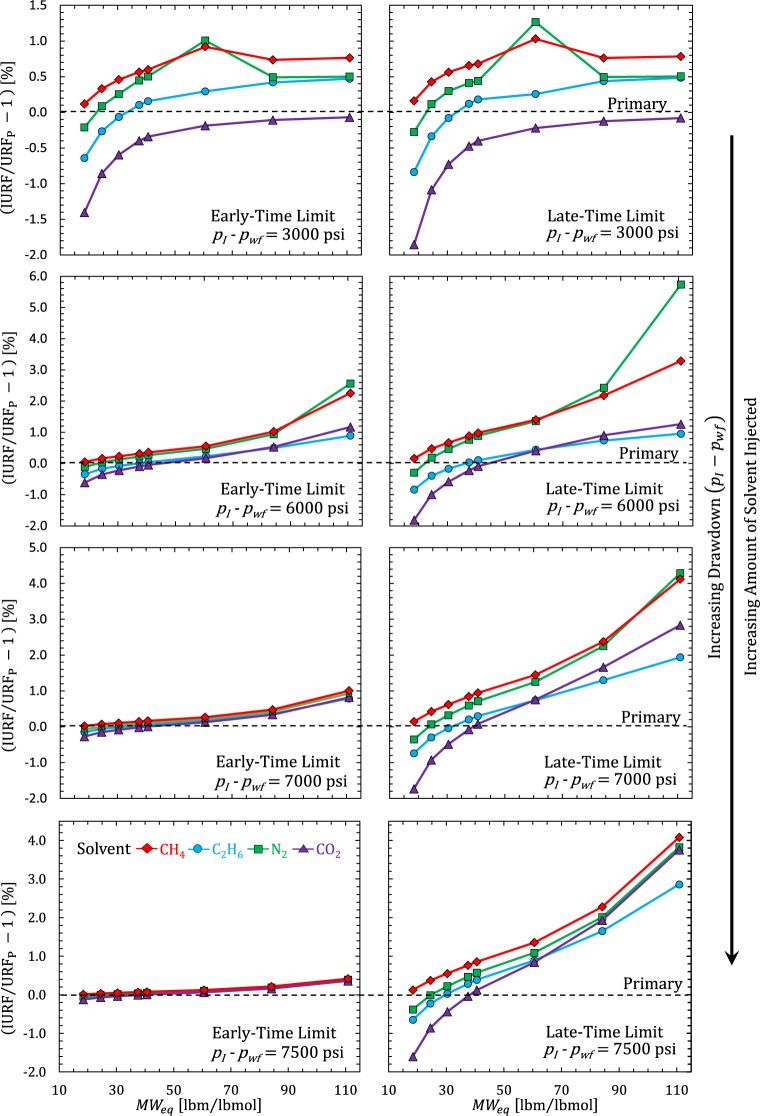
Relative RF improvement (IURF/URF_P_ − 1) [%] versus *MW*_*eq*_ [lbm/lbmol] for Fig. 9 cases.

Figure 10 shows the relative RF improvement (IURF/URF_P_ − 1) [%] for the cases in Fig. 9. According to Fig. 10, the ratio of IURF (late-time limit)/IURF (early-time limit) changed by a factor of 2 at *p*_*I*_ − *p*_*wf*_ = 6000 psi, to a factor of 4 at *p*_*I*_ − *p*_*wf*_ = 7000 psi, to a factor of 10 at *p*_*I*_ − *p*_*wf*_ = 7500 psi for oils. The small drawdown scenario (*p*_*I*_ − *p*_*wf*_ = 3000 psi) showed negligible differences between ETL and LTL IURF. This is because the changes in density and pore volume in Eqs (3) and (5) roughly cancel each other out due to the small amount of solvent injected. In contrast, dry gas and condensate were sensitive to solvent composition, with only C_1_ and N_2_ solvents providing positive IURF values over the range in *p*_*wf*_ and soak duration limits. CO_2_ and C_2_ solvents performed poorly for condensates, except perhaps very rich condensates. This makes sense because these solvents are similar to condensates. The large relative improvement in recovery using N_2_ seen in Fig. 10 for the heavy oil (*MW*_*eq*_ = 110 for *p*_*I*_ − *p*_*wf*_ = 6000 psi) and volatile oil (*MW*_*eq*_ = 60 for *p*_*I*_ − *p*_*wf*_ = 3000 psi) indicate that the solvent-hydrocarbon mixture crossed a phase envelope boundary (or changed from one side of a critical point to another).

In summary, solvent IURF is beneficial whenever hydrocarbon density is lower during the puff than primary production. The magnitude of this benefit depends on the initial fluid composition, solvent type, amount injected, pressure, temperature. The ETL and LTL equilibrium fluid compositions have very different solvent strengths (mole fraction of solvent), and thus, very different amounts of density change. Recovery in the LTL tends to be substantially larger than the ETL. The reason is that a small density change in the entire reservoir in the LTL outperforms a very large density change in a small fraction of reservoir volume in the ETL. However, Fig. 9 suggests that HnP is viable (IURF > 0) in the ETL for oils with any solvent (miscible or immiscible), provided that the fractures contained sufficient pore volume to make the effort worthwhile. Perfect mixing minimizes solvent recycling while *V*_*wf*_-only mixing maximizes it. Solvent recycling is a key concern for gas injection projects in fractured reservoirs with low permeability matrix for both technical and economic reasons^17,41^. Thus, soak duration would largely be driven by time-value of production considerations and the costs associated with obtaining solvent, recovering solvent from the produced hydrocarbon, and (re)-injection.

## Discussion

In the case of primary production, Cronin *et al*.^6^ illustrated why the ultimate recovery factor from primary production (URF_P_) is independent of hydraulic fracture spacing, matrix-fracture contact area, and matrix transport coefficients. These parameters control recovery rates, but they do not control the equilibrium hydrocarbon density that would be reached after a long time (as *t* → ∞). Vapor-like hydrocarbon fluid compressibilities are approximately 100 times greater than liquid-like hydrocarbon compositions. As a consequence, pressure depletion has a much smaller effect on reducing hydrocarbon density for single-phase oils versus gases (Figs 3, 8). While it may be poor conventional reservoir management, URF improves with the development of a gas-like second phase (L → L + V) due to depletion or compositional changes following solvent injection. For hydrocarbons close to critical points (*p*_*c*_, *T*_*c*_), small temperature changes control whether the composition behaves more liquid-like versus vapor-like since *T*_*c*_ is proportional to MW (Fig. 2). The progressive shift in the URF_P_ contours corresponds to a transition from liquid-like to vapor-like, which happens abruptly in composition (Fig. 3) and temperature space (Fig. 4).

The compositional proxy (*n*-alkane series) qualitatively predicted the observed trends for primary production in the Eagle Ford with respect to increasing drawdown, increasing temperature, and increasing equivalent MW. The compositional proxy also revealed the solvent IURF potential for heavier oils. Specifically, the compositional proxy showed solvent assisted IURF was large (Figs 5 and 6) compared to primary recovery (Fig. 3), which means that the relative recovery improvement over primary recovery alone (IURF/URF_P_) would be substantial for oils, which is precisely the behavior observed in Fig. 10 for oil compositions.

Maximizing long-term hydrocarbon recovery requires net solvent transfer deeper into matrix to compositionally dilute the hydrocarbons in place and assist with volume expansion. However, it has been clearly demonstrated at the field-scale that relatively short injection-soak-puff cycles have improved incremental oil recovery in the South Texas Eagle Ford^42,43^. Single-cycle IURF results from the early-time limit (Figs 8–10) provide evidence that these short-term improvements could be explained based solely on the rapid pressurization and production of fluid in the fractures during multiple cycles of solvent injection and puff. This is conceptually equivalent to operating the wellbore-fractures like a set of bellows that are quickly inflated and deflated during multiple cycles of solvent injection and puff. Solvent IURF calculations assumed the most pessimistic assumptions possible; uniform depletion prior to injection and no diffusive transfer between matrix and fractures for the early-time limit (ETL) scenario. In practice, diffusive influx of hydrocarbons would replenish the fractures due to the favorable concentration gradients created due to fluid expansion and compositional dilution caused by the introduction of solvent. Recall that any primary production history would establish density gradients facilitating diffusive recharge of the fractures. Thus, field conditions would be more favorable than the lower limits our calculations reflect.

Optimal cycle frequency, soak duration, and solvent injection pressure is an actively studied problem^1,7,8,11,26,42,44–47^. However, we argue that the concept of “optimal” merely reflects the designer’s criteria for maximum tolerable solvent recycling and time-value of production, neither of which changes the underlying fact that long term recovery from matrix (where hydrocarbon primarily resides) requires net solvent transfer deeper into matrix. In this way, our state-function (time-independent) solvent IURF methods provide clear guidance on problems that have previously only been addressed from time-dependent perspectives.

Perfect mixing would be impossible if geologic barriers/dislocations exist within a reservoir to prevent transport (even after an infinite time) within a particular gross rock volume. Therefore, inaccessible pore volume should be excluded from the URF calculation. We chose to assume a non-deforming reservoir with a well-connected constant pore volume when presenting the unified theory for URF (primary and solvent) to emphasize the importance of equilibrium phase behavior and simplify the presentation and scope of the study. In addition, we omitted explicit reference to the effects of compaction/consolidation (changes in porosity and/or the bulk reservoir volume), adsorption, gravity, and spatially variable reservoir properties, pressure, and temperatures. Trivial modifications to Equation (1) expand the generality of the URF procedure,

6 $$UR{F}_{i}=1-\frac{{C}_{i,F}}{{C}_{i,I}}=1-\frac{{({\iiint }_{V}[(\varphi \sum _{j=1}^{{n}_{p}}({S}_{j}{\rho }_{j}{\omega }_{ij})+(1-\varphi ){\rho }_{s}{\omega }_{is}){V}_{b}])}_{F(x,y,z)}}{{({\iiint }_{V}[(\varphi \sum _{j=1}^{{n}_{p}}({S}_{j}{\rho }_{j}{\omega }_{ij})+(1-\varphi ){\rho }_{s}{\omega }_{is}){V}_{b}])}_{I(x,y,z)}},$$

where *ρ*_*s*_ [lbm/ft^3^] is the solid (grains) density, *ω*_*is*_ [-] is the mass fraction for component *i* adsorbed in the solid phase *s*, *V*_*b*_ is bulk volume, with a volume integral over the gross reservoir volume honoring the spatial dependencies of the bracketed term at initial *I*(*x*, *y*, *z*) and final *F*(*x*, *y*, *z*) conditions with respect to composition, pressure, temperature, confinement effects on phase equilibria and properties, gravity effects, reservoir deformation, and reservoir heterogeneity. For completeness, multiple solid phase constituents (e.g. inorganic versus organic matrix domains^48^) would be incorporated by modifying the adsorption term (1 − *ϕ*) *ρ*_*s*_*ω*_*is*_ to $(1-\varphi )\sum _{k=1}^{{n}_{m}}{S}_{k}{\rho }_{k}{\omega }_{ik}$, where *S*_*k*_ [−] is the volume fraction, *ρ*_*k*_ [lbm/ft^3^] is the density, and *ω*_*ik*_ [−] is the mass fraction for component *i* adsorbed within the *k*^*th*^ solid-phase constituent and *n*_*m*_ is the total number of solid-phase constituents.

## Conclusions

We proposed an approach to unify our understanding of the ultimate recovery from primary production and solvent-based processes across the full range of hydrocarbon compositions. The unifying theory is based on equilibrium density changes between the initial and final state and practically adjustable factors such as drawdown and solvent composition/amount injected. We have drawn the following conclusions:

1. Density reduction (fluid compressibility) explains why shale oil reservoirs have extremely low recovery factor (<10%) while gas reservoirs may have recovery factors >60%, with condensates in between.
2. Ultimate hydrocarbon recovery is improved by any process that reduces mass concentration via fluid expansion or compositional dilution.
3. Equilibrium phase behavior provides a transport independent method to calculate the absolute theoretical upper-bound value for mass recovery as *t* → ∞.
4. Maximizing long-term hydrocarbon recovery requires net solvent transfer deeper into the matrix to dilute the hydrocarbons in place and assist with volume expansion (which eventually requires long soaks). Multiple huff‘n’puff cycles at field pressure will allow for greater net solvent transfer into the matrix.
5. Short term incremental recovery benefits can be attained with minimal soaking, particularly in oil compositions with enough fluid in the early-time volume (wellbore and fractures).
6. The late-time limit (LTL) and early-time limit (ETL) define the theoretical bounds for IURF as a function of how much solvent-hydrocarbon mixing occurs in the reservoir. The IURF performance of a real HnP cycle will reside between these two limits.
7. C_1_ and N_2_ were the most beneficial solvents over the entire range of fluid compositions (gas, condensates, and oils) based on equal injection pressure and equal injected volume comparisons. CO_2_ and C_2_ solvent were sensitive to producing pressure and tended to benefit only oil compositions or rich condensates at large drawdown values.

We emphasize that our path independent URF calculations do not replace traditional reservoir simulation. Instead, our calculations provide a practical means to define the maximum recovery attainable from primary production or cyclic solvent processes based on reservoir fluids and the key adjustable factors (drawdown and solvent composition/amount). Indeed, URF calculations explain the astounding recovery contrast from shale gas versus shale oil reservoirs. Although URF may not be achievable during the life-cycle of the field development and production, it provides valuable insights on the role of phase behavior on hydrocarbon recovery from ultratight reservoirs.
